# Enhanced activity of NLRP3 inflammasome in peripheral blood cells of patients with active rheumatoid arthritis

**DOI:** 10.1186/s13075-015-0775-2

**Published:** 2015-09-19

**Authors:** Christianna Choulaki, Garyfallia Papadaki, Argyro Repa, Eleni Kampouraki, Konstantinos Kambas, Konstantinos Ritis, George Bertsias, Dimitrios T. Boumpas, Prodromos Sidiropoulos

**Affiliations:** Rheumatology, Clinical Immunology and Allergy, Faculty of Medicine, University of Crete, 71003 Iraklion, Greece; Laboratory of Molecular Hematology, Democritus University of Thrace, 68100 Alexandroupolis, Greece; Division of Infections and Immunity, Institute of Molecular Biology and Biotechnology-FORTH, Nikolaou Plastira 100, 70013 Iraklion, Greece; Biomedical Research Foundation of the Academy of Athens, 4 Soranou Ephessiou, 115 27 Athens, Greece; 4th Department of Medicine, Rheumatology and Clinical Immunology, National and Kapodistrian University, Athens, Greece

## Abstract

**Introduction:**

Interleukin-1β (IL-1β) is a major inflammatory cytokine, produced predominantly by innate immune cells through NLRP3-inflammasome activation. Both intrinsic and extrinsic danger signals may activate NLRP3. Genetic variations in NLRP3-inflammasome components have been reported to influence rheumatoid arthritis (RA) susceptibility and severity. We sought to assess the activity of NLRP3-inflammasome in patients with active RA compared to healthy individuals.

**Method:**

Intracellular protein expression of NLRP3, ASC, pro- and active caspase-1, pro- and active IL-1β was assessed by immunoblotting both at baseline and upon inflammasome activation. NLRP3 function (IL-1β secretion) was assessed upon priming of TLR2 (Pam(3)CysSK(4), TLR3 (poly(I:C)) or TLR4 (LPS) and ATP sequential treatment. We used caspase inhibitors (casp-1, 3/7 and 8) to assess their contribution to IL-1β maturation. All experiments were performed in whole blood cells.

**Results:**

Active RA patients (n = 11) expressed higher basal intracellular levels of NLRP3 (p < 0.008), ASC (p < 0.003), active caspase-1 (p < 0.02) and pro-IL-1β (p < 0.001). Upon priming with TLR4 (LPS) and ATP, RA-derived cell extracts (n = 7) displayed increased expression of NLRP3 (p < 0.01) and active caspase-1 (p < 0.001). Secreted IL-1β in culture supernatants from whole blood cells activated with TLR4 (LPS) or TLR3 agonist (poly(I:C)) plus ATP was higher in RA patients (n = 20) versus controls (n = 18) (p < 0.02 for both). Caspase-1 inhibition significantly reduced IL-1β secretion induced by all stimuli, whereas caspase-8 inhibition affected only TLR4 and TLR3 cell priming.

**Conclusion:**

Patients with active RA have increased expression of NLRP3 and NLRP3-mediated IL-1β secretion in whole blood cells upon stimulation via TLR3 and TLR4 but not TLR2. In these patients, IL-1β secretion seems to be predominately driven by caspase-1 and caspase-8. Targeting NLRP3 or downstream caspases may be of benefit in suppressing IL-1β production in RA.

## Introduction

Inflammasomes are cytosolic multiprotein complexes that drive the production of inflammatory cytokines, mainly interleukin-1β (IL-1β) and IL-18, in response to pathogens or danger signals [1]. Inflammasomes are composed of a danger sensor, an adaptor protein that is mainly the apoptosis-associated speck-like protein containing a CARD (ASC protein), and caspase-1. There is remarkable diversity in the sensor proteins that form the inflammasomes, thus leading to the assembly of distinct complexes specialized to sense various signals. The nucleotide binding domain and leucine-rich repeat pyrin 3 domain (NLRP3 or cryopyrin) sensor protein, organizes the assembly of the best-characterized inflammasome, the NLRP3-inflammasome. A diverse set of signals has been shown to activate NLRP3, including pathogens, danger signals from damaged/stressed cells and environmental microparticles [2]. NALP3-inflammasome assembly results in ASC polymerization, procaspase-1 self-activation to the active protease, which then catalyzes pro-IL1β maturation [3]. Mutations in the gene encoding for NLRP3 have been linked to autoinflammatory syndromes (cryopyrinopathies) associated with aberrant IL-1β production [4], which have been further characterized by mouse models of mutant NLRP3 [5, 6].

Rheumatoid arthritis (RA) is a chronic inflammatory arthritis, which may be caused by various combinations of genetic and environmental triggers. IL-1β drives cartilage destruction, while its role in disease pathogenesis is further supported by the efficacy of IL-1β blockade in mouse and humans [7]. Interestingly, mutations in NLRP3-inflammasome proteins (NLRP3 and CARD8) have been associated with RA susceptibility and severity in some but not all ethnic backgrounds [8–10]. Animal studies have revisited the role of NLRP3 in the evolution of arthritis. Although earlier studies in the collagen- and antigen-induced arthritis models suggest that disease pathology is ASC-related but independent of NLRP3 [11, 12], more recent studies in a novel spontaneous arthritis model (A20^myel-KO^ mice) show that arthritis pathology critically relies on the NLRP3 inflammasome/IL-1β axis [13]. P2X7 purinergic receptor (P2X7R) an ATP-gated ion channel is an important cell surface inducer of key inflammatory cytokines mainly through NLRP3 activation [14]. Its role in inflammatory responses of RA has been shown in the animal model of arthritis [15] and recently, a P2X7R antagonist has been evaluated in a clinical study [16].

Although there is emerging evidence for involvement of the NLRP3-inflammasome/IL-1β axis in the inflammatory responses of RA, studies in humans are scarce [17, 18]. We therefore sought to investigate in peripheral blood cells the expression of NLRP3-related proteins, their potential to promote IL-1β maturation and secretion upon stimulation. Our results demonstrate enhanced expression and function of NLRP3-iflammasome in the peripheral blood of active RA patients.

## Methods

### Patients and treatment

Peripheral blood samples were obtained from patients diagnosed with RA according to the 1987 American College of Rheumatology (ACR) criteria [19], who were followed by the Rheumatology Clinic of the University Hospital of Crete. At the time of sampling all patients had high disease activity according to the disease activity score based on the 28 joint counts (DAS28) [20]. The study was approved by the Ethics Committee of the University Hospital of Heraklion, University of Crete School of Medicine, and all participants gave informed consent.

### Ex vivo activation of NLRP3-inflammasome in whole blood cells

Although human innate and adaptive immune responses are typically studied in vitro in isolated peripheral blood mononuclear cells (PBMCs), this approach has intrinsic limitations and therefore assays based on whole blood have been proposed [21] and lately better standardized to assess immune function in humans [22]. Moreover, although the NLRP3-inflammasome is expressed predominantly in monocytes/macrophages and dendritic cells, neutrophils, the most abundant cells in peripheral blood, also express functional NLRP3-inflammasome [23]. Thus, we performed all experiments in whole blood cells.

Heparin-anticoagulated whole blood from RA patients and healthy controls was collected and immediately processed. Cell viability assessed by trypan blue staining was >90 % and did not differ between patients and healthy individuals. After red blood cell (RBC) lysis in ammonium persulfate buffer (NH_4_Cl 0.15 M/KHCO_3_ 10 mM/Na_2_EDTA 0.1 mM, pH 7.2 − 7.4) baseline expression of NLRP3, IL-1β, caspase-1 was assessed by immunoblotting in cell lysates.

NLRP3-inflammasome function (activation) was assessed in cultures (1.5 × 10^6^/mL) at 37 °C (5 % CO2) in RMPI-1640 (Gibco Thermofisher Scientific, Waltham, MA. USA) medium supplemented with 10 % heat-inactivated FBS (Gibco Thermofisher Scientific Waltham, MA. USA) with or without pre-treatment with various Toll-like receptor (TLR) ligands, followed by ATP pulse to activate NLRP3-inflammasome (ATP 5 mM, 20 min) (Sigma-Aldrich Chemie GmbH, Buchs, Switzerland, #A1852). In detail, TLR ligands that were applied were for TLR4 (lipopolysaccharide (LPS), 250 pg/mL, 2 h) (Calbiochem Billerica, MA, USA, #437627), TLR3 (Polyinosinic-polycytidylic acid, 50 μg/mL, 2 h) (Sigma-Aldrich Chemie GmbH, Buchs, Switzerland, #P0913) and TLR2 (pam3CysSK 200 ng/mL, 2 h) (EMC microcollections Tübingen, Germany, #L2000). Caspase-1 inhibitor Ac-YVAD-CHO (10 μM, 15 min) (Calbiochem Billerica, MA, USA #400010), or caspase-8 inhibitor (Calbiochem Billerica, MA, USA #218840)  or caspase-3/7 inhibitor (Calbiochem Billerica, MA, USA, #218832), were applied for 15 min prior to ATP pulse. Cell culture supernatants were collected prior to and following ATP treatment to measure levels of TNFα and IL-1β by ELISA (e Bioscience San Diego, CA, USA, #88-7346-22 and 88-7010-88 respectively)  and cell extracts were collected to assess intracellular NLRP3-inflammasome activation.

### Western blot analysis

Intracellular expression of NLRP3, caspase-1, and IL-1β was assessed by immunoblotting in lysates from freshly isolated whole blood cells and also from whole blood cells cultured in medium alone or in the presence of TLR ligands/ATP as described above. Cells were lysed in 10 mM Tris–HCl pH 8.0, 150 mM NaCl, 1 mM EGTA, 1 % Triton-X 100 in the presence of complete mini EDTA-Free Protease Inhibitor Cocktail (Roche, Basel, Switzerland, #04693124001). Protein concentration was determined by the Bradford method using the Bio-Rad protein assay solution (Bio-Rad Laboratories GmbH Hercules CA, USA, #500-006) Lysates were subjected to SDS-PAGE using 12 % polyacrylamide gels, and were subsequently transferred to 0.2 μm nitrocellulose membrane. The membrane was blocked for 1 h at room temperature followed by incubation overnight at 4 °C with antibodies against human NALP3 (Alexis Biocehmicals Enzo Life Sciences, Inc Farmingdale, NY, USA, #ALX-804-819), caspase-1 (Cell Signaling technologies Danvers, MA, USA, #2225), anti-IL-1β (Cell Signaling technologies Danvers, MA, USA, #2022), anti-ASC (Santa Cruz biotechnology Dallas, Texas U.S.A., #sc-271054)  and β-actin (Merck-Milipore Billerica, MA, USA, #MAB1501). Afterwards, the blots were washed and incubated with horseradish peroxidase-conjugated secondary antibodies for 1 h at room temperature. The blots were developed with electro-chemiluminescence using ECL plus (Pierce-Thermofisher Scientific Waltham, MA, USA). Protein densitometry was assessed using Image J and the intensity of the protein of interest was normalized to β-actin expression.

### Enzyme-linked immunosorbent assay (ELISA)

IL1-β and TNFα content in supernatants was determined by ELISA (Ebioscience-, San Diego, CA, #88-7010-88 and #88-7346-22 respectively) according to the manufacturer’s instructions.

### Statistical analysis

Results are shown as mean ± standard error (SEM). Differences between groups were analyzed by independent or paired samples *t* test as appropriate, using the Statistical Package for Social Sciences software version 20.0 (SPSS, Inc.). *P* value ≤0.05 (two-tailed) were considered statistically significant.

## Results

### Patients’ clinical characteristics at baseline

At the time of sampling, all patients had highly active disease (mean (SEM) DAS28 5.2 (0.2)) with high inflammatory serum markers (erythrocyte sedimentation rate (ESR) 39.8 (7.3) mm/h, C-reactive protein (CRP) 2.4 (1.3) mg/dL), irrespective of background treatment (Table 1).

**Table 1:** Baseline demographics and clinical characteristics of the RA patients included in the study

	RA patients (*n* = 23)
Demographics	
Age, years	52.6 (2.8)*
Gender, % female	77
Clinical characteristics	
Disease duration, years	4.7 (1)
Rheumatoid factor and/or anti-CCP positive, %	30
Disease activity score in 28 joints	6 (0.2)
Swollen (28)	8.7 (1.2)
Tender (28)	10.8 (1.7)
C-reactive protein, mg/dL	2.4 (1.3)
Erythrocyte sedimentation rate, mm/h	39.8 (7.3)
Treatments	
Naïve to treatment, n/total (%)	13/23 (56 %)
Methotrexate, n/total (%)	7/23 (30)
Methotrexate dose, mg/week	17.1 (1.5)
Leflunomide, n/total (%)	3/23 (13)
Biologic agents, n/total (%)	4/23 (17)
Glucocorticoids, n/total (%)	5/23 (22)
Glucocorticoid dose, mg/day	8.2 (3.3)

*Except where indicated otherwise, values are the mean (standard error of the mean). *anti-CCP* anti-cyclic citrullinated peptide

### Freshly isolated peripheral blood from active RA expresses increased levels of NLRP3-inflammasome proteins

**Fig. 1:**
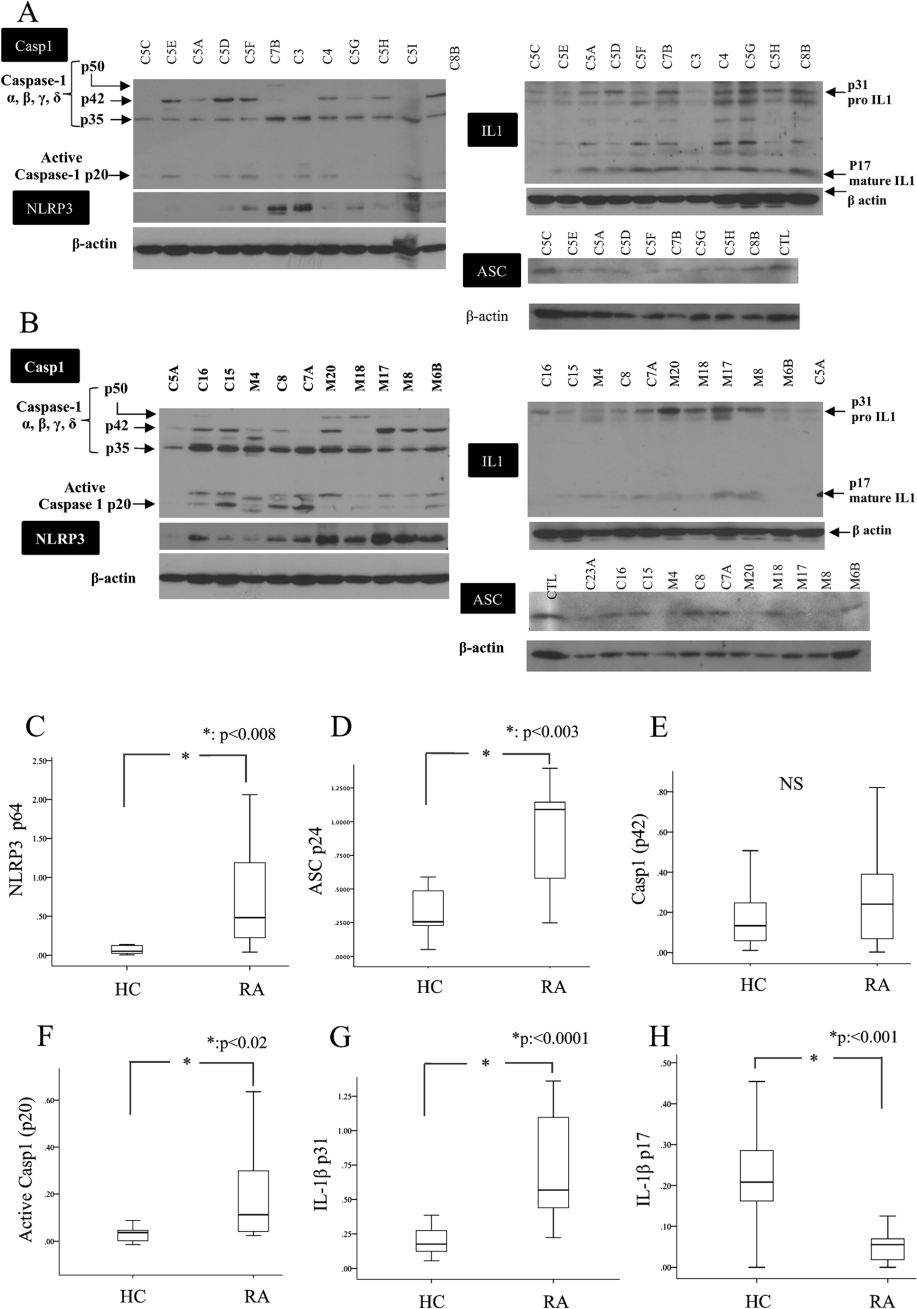
Baseline expression of nucleotide binding domain and leucine-rich repeat pyrin 3 domain (*NLRP3*) inflammasome-related proteins in controls (**a**) as compared to patients with rheumatoid arthritis (*RA*) (**b**). Western blots were performed in cell lysates of freshly isolated whole blood cells for CASP 1, NLRP3, IL1β and ASC, from healthy controls (**a**) (n = 11) and patients with active RA (**b**) (n = 11). Protein densitometry was assessed by the use of Image J program and the intensity of the protein of interest was normalized to β-actin expression (**c**-**h**). *HC* healthy controls, *CTL* control

First we assessed the baseline expression of NLRP3-inflammasome-related proteins in RA patients compared to healthy controls. To this end we performed immunoblotting in freshly isolated unstimulated whole blood cells and found that patients with active RA (n = 11) expressed higher intracellular levels of NLRP3 (*p* <0.008), ASC (*p* <0.003) and active caspase-1 (*p* <0.02) as compared to controls (n = 11) (Fig. 1a, b). Accordingly, RA patients expressed higher intracellular levels of pro-IL-1β (*p* <0.0001), while levels of active IL-1β were lower compared to controls (*p* <0.001) (Fig. 1c-h). As expected, mature IL-1β levels in cell culture supernatants were minimal and comparable between patients and controls (data not shown).

### Activation of NLRP3-inflammasome in a whole blood cell assay

**Fig. 2:**
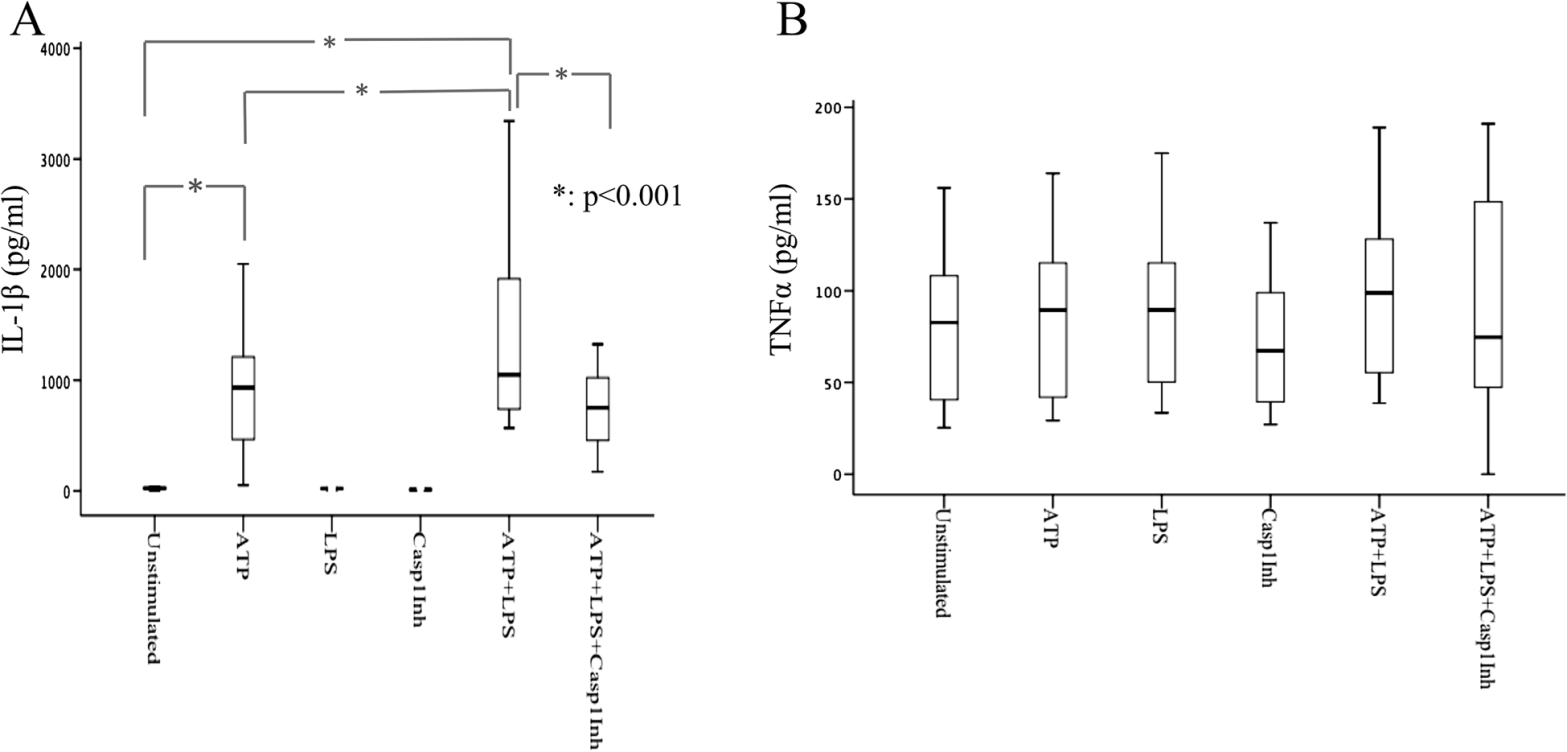
Specificity for nucleotide binding domain and leucine-rich repeat pyrin 3 domain (NLRP3) stimulation of the method applied. **a** Toll-like receptor (TLR)4 priming (ultra-pure lipolysaccharide (*LPS*) (250 pg/mL, 2 h)) followed by ATP pulse (5 mM, 20 min) efficiently activates NLRP3-inflammasome, assessed by IL-1β secretion in supernatants of whole blood cells of healthy controls (n = 15). **b** No increase in TNFα secreted by peripheral whole blood cells stimulated by the same triggers (n = 12). *HC* healthy controls, *RA* rheumatoid arthritis, *Casp1Inh* caspase1 inhibitor

Optimal NLRP3-inflammasome activation requires two signals. The first one is an inflammatory stimulus that signals through the TLRs or cytokine receptors, to upregulate gene products required for activation of the caspase-1, and the second is an NLRP3-specific trigger, typically ATP [24]. In preliminary experiments performed in blood drawn from healthy individuals (n = 15), we assessed the efficiency and specificity of sequential TLR4 and ATP activation to induce NLRP3. TLR4 priming (ultra-pure LPS, 250 pg/mL, 2 h) followed by pulse ATP stimulation (5 mM, 20 min) efficiently activated NLRP3-inflammasome, assessed by IL-1β secretion in supernatants, as compared to either stimulus alone (*p* <0.001) (Fig. 2a). IL-1Ra belongs to the IL-1 family of cytokines, and is also produced upon LPS stimulation [25]. LPS and ATP sequential treatment effectively induced secreted IL-1Ra (*p* = 0.0001 vs unstimulated cells, data not shown). In contrast, we observed no increase in TNFα secretion upon combined LPS and ATP treatment, supporting the specificity of the assay for NLRP3-inflammasome induction (Fig. 2b). Addition of a caspase-1 inhibitor effectively inhibited IL-1β secretion (Fig. 2a).

### Activation of NLRP3-inflammasome results in higher IL-1β secretion by peripheral blood cells in active RA

**Fig. 3:**
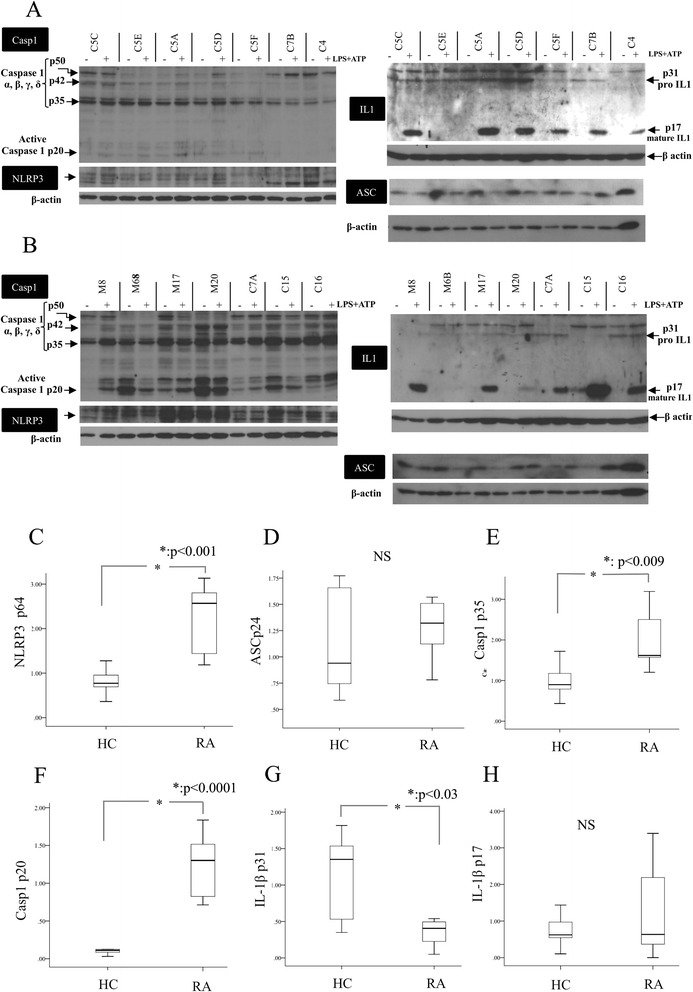
Induction of nucleotide binding domain and leucine-rich repeat pyrin 3 domain (*NLRP3*)-inflammasome related proteins in peripheral whole blood cells, upon sequential stimulation with lipolysaccharide (*LPS*) (250 pg/mL, 2 h) and ATP (5 mM, 20 min), in healthy individuals and patients with rheumatoid arthritis (*RA*). Western blots were performed in freshly isolated whole blood cells without (−) or with (*+*) LPS + ATP treatment from healthy controls (**a**) (n = 7) and patients with active RA (**b**) (n = 7). Protein densitometry was assessed using Image J program and the intensity of the protein of interest was normalized to that of β-actin (**c-h**). *HC* healthy controls

We have shown that NLRP3-inflammasome-related proteins are overexpressed in freshly, unstimulated peripheral blood cells from RA patients with active disease. Next we sought to assess whether upon its activation, NLRP3-inflammasome has differential expression and function in RA. For this, we applied sequential treatment with TLR4 ligand (LPS) and ATP on whole blood cells from healthy controls (n = 7, Fig. 3a) and patients with active RA (n = 7, Fig. 3b) and quantified intracellular expression of NLRP3-inflammasome-related proteins. The expression of both NLRP3 and active caspase-1 (p20) was induced upon NLRP3 induction in whole blood cells of patients as compared to controls (Fig. 3). Of interest, active IL-1β intracellular expression was comparable between the two groups.

Besides TLR4 triggering, investigators have applied other TLR ligands as the first signal for NLRP3-inflammasome activation [26]. In addition to TLR4 [27], signaling through TLR2 [28] and TLR3 [29] may participate in inflammatory responses in RA. To assess NLRP3-inflammasome function upon activation with stimuli relevant to RA, we applied sequential treatment of whole blood cells with TLR2, TLR3 or TLR4 ligands followed by ATP [26] and measured IL-1β.

**Fig. 4:**
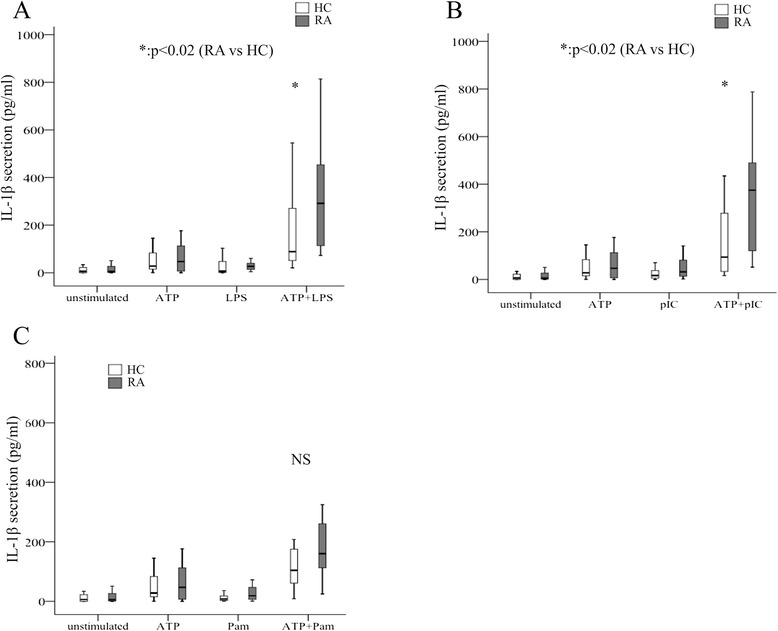
Functional assessment of nucleotide binding domain and leucine-rich repeat pyrin 3 domain (NLRP3)-inflammasome upon activation. Whole blood from patients (n = 20) or healthy individuals (n = 18) were primed with Toll-like receptor (TLR)4 (lipopolysaccharide (*LPS*), 250 pg/mL, 2 h) (**a**), TLR3 (poly IC, 50 μg/mL, 2 h) (**b**) or TLR2 (pam3CysSK 200 ng/mL, 2 h) (**c**) ligands and sequentially pulsed with ATP (ATP 5 mM, 20 min). IL-1β secretion in cell supernatants was quantified by ELISA (pg/mL). *HC* healthy controls, *RA* rheumatoid arthritis, *pIC* polyinosinic-polycytidylic acid, *Pam* pam3CysSK

We found that upon TLR2, TLR3 or TLR4 cell priming and sequential ATP-pulse, high levels of IL-1β were secreted as compared to either stimulus alone (Fig. 4). Interestingly, cells from RA patients (n = 20) secreted significantly larger amounts of IL-1β compared to controls (n = 18) upon cell priming with TLR3 or TLR4 ligands (Fig. 4a, b), while IL1-β secretion was comparable when TLR2 ligand was applied (Fig. 4c). These data, together with the findings of higher intracellular NLRP3 and active caspase-1 upon NLRP3-inflammasome induction in whole blood cells in RA, indicate increased function of NLRP3-inflammasome in RA patients.

### Different caspases are involved in pro-IL1β maturation

**Fig. 5:**
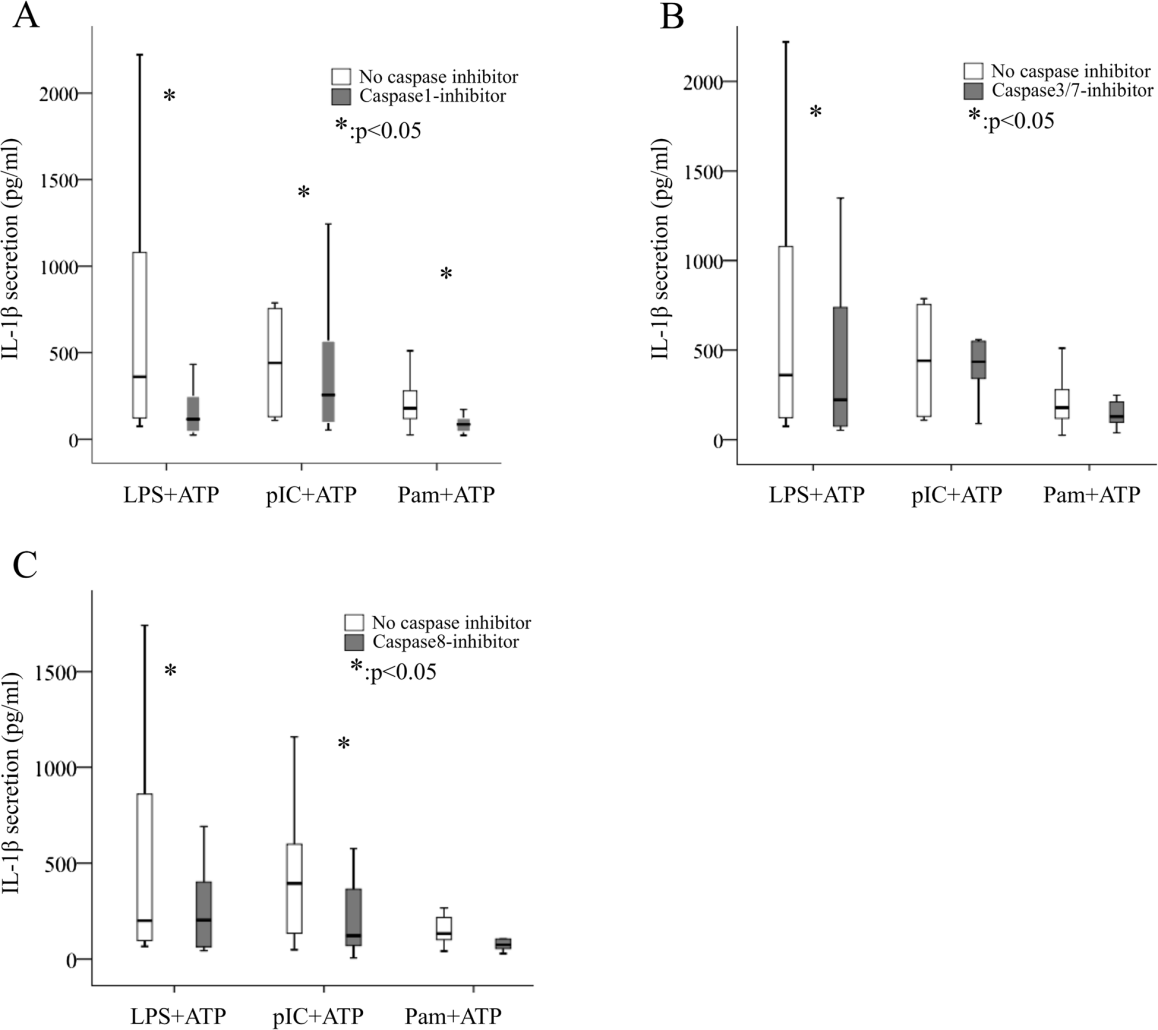
Assessment of the effect of inhibition of various caspases in IL-1β maturation upon nucleotide binding domain and leucine-rich repeat pyrin 3 domain (NLRP3) activation. Whole blood cells from patients with rheumatoid arthritis (RA) (n = 20) were primed with Toll-like receptor (TLR)4 (lipopolysaccharide (*LPS*), 250 pg/mL, 2 h) (**a**), TLR3 (poly IC, 50 μg/mL, 2 h) (**b**) or TLR2 (pam3CysSK 200 ng/mL, 2 h) (**c**) ligands and sequentially pulsed with ATP (ATP 5 mM, 20 min). Inhibitors of caspase-1 (**a**) or caspase-3/7 (**b**) or caspase-8 (**c**) were applied. IL-1β secretion in cell supernatants was quantified by ELISA (pg/mL). *pIC* polyinosinic-polycytidylic acid, *Pam* pam3CysSK, *Casp1Inh* caspase1 inhibitor, *Casp3/7Inh* caspase3 and 7 inhibitor, *Casp8Inh* caspase8 inhibitor

Upon NLRP3 activation, pro-caspase-1 is auto-cleaved to its active form to induce proteolysis of pro-IL-1β. Nevertheless, there is evidence that inflammatory caspases other than caspase-1 may also contribute to IL-1β activation [30–32]. Thus, we sought to assess the contribution of other caspases (caspase-3/7, caspase-8) in NLRP3-mediated IL-1β production in RA. To this end, we applied caspase-1, caspase-3/7 and caspase-8 inhibitors prior to ATP pulse and measured secreted IL-1β (see “Methods”). All three caspase inhibitors inhibited IL-1β secretion (Fig. 5). Interestingly, only caspase-1 inhibition significantly reduced IL-1β secretion by whole blood cells from patients and across all three TLR agonists used for cell priming (*p* <0.05). On the other hand, both caspase-3/7 and caspase-8 inhibition reduced IL-1β secretion, only upon TLR4 cell priming, while caspase-8 inhibited IL-1β secretion upon TLR3 priming as well.

## Discussion

Herein we sought to characterize the expression and function of NLRP3-inflammasome in RA. Compared to healthy individuals, whole blood cells from RA patients express higher basal amounts of NLRP3-related proteins and they demonstrate even higher expression following NLRP3 activation. Importantly, RA whole blood cells were found to secrete larger amounts of IL-1β upon NLRP3 activation, supporting that the overexpressed NLRP3-inflammasome is also overactive. These findings support a role of this intracellular complex in the systemic inflammation of RA.

In the context of sterile inflammation such as in the case of RA, multiple endogenous signals have been shown to induce NLRP3-inflammasome expression and activation. Inflammatory cytokines like TNFα and IL-1β [33, 34], several danger-associated molecular patterns (DAMPs) like ATP released by mitochondria of damaged cells [35], HMGB1 [36] and S100A proteins [37]. Interestingly, many of these DAMPs are upregulated in peripheral blood in RA [38, 39]; they may participate through inflammasome activation in the systemic inflammation of the disease and some have been assessed as therapeutic targets for inflammatory arthritis [40]. Our data corroborate recently reported data suggesting upregulation of NLRP3-inflammasome-related gene expression in mononuclear cells (PBMCs) in peripheral blood from patients with active RA [17] and earlier data showing increased NALP3 mRNA levels in the synovium in RA compared with osteoarthritis (OA) [18]. Clinicians are familiar with the limited efficacy of IL-1Ra (anakinra) in RA patients. This has been attributed to factors such as limited bioavailability (due to rapid renal clearance) and the need to block the majority of cellular IL-1R in order to effectively inhibit the effects of IL-1β [41]. Thus, elucidating the molecular components and intracellular cascades that control IL-1β production, are an alternative for interfering with IL-1β-mediated effects aiming to inhibit its production. Several small-molecule inhibitors of NLRP3-inflammasome have been described and already tested in vitro or in animal models of systemic inflammatory disease [42]. Whether this approach will have better clinical results as compared to IL-1Ra remains to be seen.

In the collagen- and antigen-induced arthritis mouse models, pathology has been shown to be independent of NLRP3 but dependent on ASC [11, 12]. Recently, Vande Walle et al*.* have shown that arthritis pathology is critically related to the NLRP3 inflammasome/IL-1 signaling axis, in the arthritis model *A20*^*myel-KO*^ mice, in which the *A20/Tnfaip3* RA susceptibility gene is deleted in myeloid cells [13]. This new model *A20*^*myel-KO*^ might be suitable for validation of therapies targeting NLRP3-inflammasome or IL-1 signaling. Animal models of inbred mice have not, in most cases, been a reliable guide to developing treatments for human immunological diseases and there is also significant reservation about their relevance to the initiation of RA and the chronic inflammatory processes of RA.

Addressing the involvement of inflammasome in human disease has been challenging. Demonstrating IL-1β cleavage by caspase-1 in biologic specimens is difficult, while demonstrating increased activity of the inflammasome is even more challenging [43]. We applied immunoblotting for NLRP3-inflammasome-related protein assessment intracellularly and we quantified IL-1β secretion in peripheral blood. Although both have limitations, mainly when addressing inflammasome function, nevertheless they have been applied in most of the human studies so far [44]. Recently, it has been shown that in mouse bone-marrow-derived macrophages (BMDMs), the activation of inflammasomes resulted in the extracellular release of active inflammasome oligomers as particles that acted extracellularly as danger signals to amplify the inflammatory response by activating caspase-1 [45]. Thus, our method of assessment of intracellular inflammasome-related proteins may underestimate its presence and function.

Although monocytes, macrophages and dendritic cells are the major NLRP3-expressing cells, human and mouse neutrophils have been also shown to have functional NLRP3-inflammasome [23]. Neutrophils are the most abundant cells in peripheral blood. Although human immune responses are usually studied in vitro in isolated PBMCs, assays based on whole blood cells have been proposed and lately have been better standardized, to assess immune function in humans [22]. Notably, direct measurements made in whole blood have the advantage of minimizing contamination and sample handling. Moreover, maintaining total leukocytes (e.g., polymorphonuclear cells) and platelets in a plasma matrix may provide a more accurate reflection of in vivo responsiveness to immune stimuli [22]. In preliminary experiments, we applied the same triggers in peripheral blood isolated monocytes and we found comparable results to those of whole blood. For these reasons, we performed our experiments in whole blood after red cell lysis.

Caspase-1 is an inflammatory caspase activated by the assembly of inflammasomes [46]. It is a protease that controls the release of various leader-less proteins, as well as its own release [47]. Interestingly, this is not the only protease involved in IL-1β maturation [30–32]. Caspase-4 has been shown to participate in IL-1β maturation probably through caspase-1 activation [32]. Moreover, a role in canonical and noncanonical NLRP3-inflammasome priming and activation through TLRs has been attributed to caspase-8 [30, 31]. In our functional studies we applied selective caspase inhibitors to assess their importance in IL-1β secretion. Although all caspase inhibitors applied could downregulate IL-1β secretion upon TLR priming and ATP pulse, only caspase-1 inhibitor resulted in significant downregulation of IL-1β with all three different stimuli (TLR2, TLR3 and TLR4) applied. Caspase-8 inhibitor significantly downregulated IL-1β secretion only upon TLR4 and TLR3 cell priming. These data support the role of caspase-1 but also of caspase-8 in inflammatory responses through NLRP3 in humans with inflammatory arthritis, as it has been shown in the aforementioned studies in mouse-derived cells and keratinocytes. The role of caspase-3 and caspase-7 in inflammatory response has not yet been established as it has been for apoptosis [48].

## Conclusion

In summary we found increased expression and function of NLRP3-inflammasome in patients with RA by applying whole blood cell analysis. These data support a role of NLRP3 in mediating systemic inflammatory responses in the context of RA. Caspase-1, and also caspase-8, may be important mediators for IL-1β release, and hence potential therapeutic targets.

## Abbreviations

ACR: American College of Rheumatology
ASC: apoptosis-associated speck-like protein containing a CARD
ATP: adenosine triphosphate
CARD8: Caspase recruitment domain family, member 8
CRP: C-reactive protein
DAMP: danger-associated molecular pattern
DAS28: disease activity score in 28 joints
ELISA: enzyme-linked immunosorbent assay
ESR: erythrocyte sedimentation rate
FBS: fetal bovine serum
HMGB1: High-mobility group protein B1
IL-1β: interleukin-1β
IL-1Ra: IL-1 receptor antagonist
LPS: lipopolysaccharide
NLRP3: nucleotide binding domain and leucine-rich repeat pyrin 3 domain
P2X7R: P2X7 receptor
PBMC: peripheral blood mononuclear cell
poly(I:C): polyinosinic:polycytidylic acid
RA: rheumatoid arthritis
RPMI: Roswell Park Memorial Institute
TLR: Toll-like receptor
TNFα: tumor necrosis factor α
